# Quantifying Time-Frequency Co-movement Impact of COVID-19 on U.S. and China Stock Market Toward Investor Sentiment Index

**DOI:** 10.3389/fpubh.2021.727047

**Published:** 2021-09-10

**Authors:** Rui Nian, Yijin Xu, Qiang Yuan, Chen Feng, Amaury Lendasse

**Affiliations:** ^1^School of Electronic Engineering, Ocean University of China, Qingdao, China; ^2^Harvard Medical School, Harvard University, Cambridge, United States; ^3^Suning Software Technology Co., Ltd., Nanjing, China; ^4^College of Technology, University of Houston, Houston, TX, United States

**Keywords:** COVID-19, co-movement, time-frequency analysis, SSEC, SZI, DJIA, SPX, NDX

## Abstract

The worldwide spread of COVID-19 dramatically influences the world economic landscape. In this paper, we have quantitatively investigated the time-frequency co-movement impact of COVID-19 on U.S. and China stock market since early 2020 in terms of daily observation from National Association of Securities Dealers Automated Quotations Index (NDX), Dow Jones Industrial Average (DJIA), Standard & Poor's 500 Index (SPX), Shanghai Securities Composite Index (SSEC), Shenzhen Securities Component Index (SZI), in favor of spatiotemporal interactions over investor sentiment index, and propose to explore the divisibility and the predictability to the volatility of stock market during the development of COVID-19. We integrate evidence yielded from wavelet coherence and phase difference to suggest the responses of stock market indexes to the COVID-19 epidemic in a long-term band, which could be roughly divided into three distinguished phases, namely, 30–75, 110–150, and 220–280 business days for China, and 80–125 and 160–175 after 290 business days for the U.S. At the first phase, the reason for the extreme volatility of stock market mainly attributed to the sudden emergence of the COVID-19 epidemic due to the pessimistic expectations from investors; China and U.S. stock market shared strongly negative correlation with the growing number of COVID-19 cases. At the second phase, the revitalization of stock market shared strong simultaneous moves but exhibited opposite responses to the COVID-19 impact on China and U.S. stock market; the former retained a significant negative correlation, while the latter turned to positively correlated throughout the period. At the third phase, the progress in vaccine development and economic stimulus began to impose forces to stock market; the vulnerability to COVID-19 diminished to some extent as the investor sentiment indexes rebounded. Finally, we attempted to initially establish a coarse-grained representation to stock market indexes and investor sentiment indexes, which demonstrated the homogenous spacial distribution in the vectorgraph after normalization and quantization, implying the strong consistency when filtering the frequent small fluctuations during the evolution of the COVID-19 pandemic, which might help insights into the prediction of possible status transition in stock market performance under the public health issues, potentially performing as the quantitative references in reasonably deducing the economic influences.

## Introduction

The global outbreak of coronavirus disease-2019 (COVID-19) has greatly impacted the world economy since early 2020. Such kind of a notable public health event can be seen as a black swan event, which brings unpredictable and unusual forces in the context of economics, and might hereby typically result in a chain of adverse market reactions and disruptions.

As the two largest economies of the world, the economic progress of both U.S. and China after the spread of the COVID-19 pandemic has particularly received worldwide attention. Up to now, from the COVID-19 live map trackers from Johns Hopkins University, we could see that almost 16.33 million people worldwide have been identified as COVID-19 cases, accounting for 5% of the total population of the world, with 3.38 million deaths. Among them, China, the first country to be hit by coronavirus, has a cumulative case count of 105,318 and a cumulative death toll of 4,860, accounting for 0.14% of the deaths of the world. The U.S. catches up with the most rapid development of coronavirus later, with a cumulative total of 3.4 million confirmed cases, accounting for 20.68% of the confirmed cases of the world and 10.2% of its total national population.

The increase of COVID-19 cases definitely exerts influencing forces onto both U.S. and China stock market. Zhang et al. (1) emphasized the pattern change of global stock market spillover since the COVID-19 outbreak, aiming to map the general pattern of country-specific risks and systemic risks in global financial market by analyzing the potential consequences of policy intervention. Baker et al. (2) argued that the COVID-19 pandemic had impacted the stock market forcefully and evaluated potential explanations for the unprecedented stock market reaction. Onali (3) studied the impact of COVID-19 cases and deaths on U.S. stock market, allowing for changes in trading volume and volatility expectations, and found the negative impact of the number of reported cases for China on U.S. stock market returns by the GARCH (1, 1) model, the negative impact of the number of reported deaths in Italy and France by VAR models, and the increased magnitude of the negative impact of VIX (Cboe volatility index) on stock market returns by Markov-Switching models. A study by Hong et al. (4) investigated the relationship between COVID-19 and the instability of both stock return predictability and price volatility in U.S., reported that a highlighted break in return predictability and price volatility of both SPX and DJIA during the COVID-19 outbreak and crisis is associated with market inefficiency, creating profitable opportunities for traders and speculators. Drake (5) explored the gold-stock market relationship in the context of COVID-19 by computing rolling correlations over time and utilizing Granger causality tests, demonstrating that the relationship was affected by the real interest rate and stock market volatility. Liu et al. (2020) explored the interaction between COVID-19, U.S. crude oil market, and stock market by a time-varying parametric vector autoregressive model, showing there was a negative association between crude oil returns and inventory returns, while COVID-19 might have a positive impact on the crude oil market and the stock market. Ashraf (6) applied the panel data analysis technique into the daily COVID-19 confirmed cases, death and stock market returns, and found that stock markets responded negatively to the growth in COVID-19 confirmed cases, which reacted more proactively compared to the number of deaths. Mazur et al. (7) investigated the performance of U.S. stock market during the crash triggered by COVID-19 and found that, during periods of large stock swings, underperforming stocks exhibited extreme asymmetric volatility that is negatively correlated with stock returns. Yousfi et al. (8) investigated the impact of the two waves of the COVID-19 pandemic on U.S. stock market and the uncertainty through a dynamic correlation approach, and argued that there was a long-term and persistent relationship between U.S. market and COVID-19, with higher volatility spillover than before. Guo et al. (9) explored connections of global stock markets during the financial crises or risks with the emphasis on COVID-19 and constructed the topological analysis of complex networks through persistence landscapes for 40 countries/regions, showing closer connections among the markets when COVID-19 spread worldwide. Chevallier (10) empirically documented the contagion of the COVID-19, tied to high-frequency data of 31 stock markets, and found that cross-border financial system interactions might enhance global financial distress, as vulnerability from one economy was transmitted to other financial systems, implying the cataclysmic impact of the COVID-19 pandemic.

Meanwhile, the hesitation and the contradiction from the investors have also been severely affected by the virus accordingly, which made the expectation of the investors quickly devastate, with the unpredictable consequences. The cognitive uncertainty about COVID-19 has occurred at the beginning, together with the limitation of the information channels, namely technical information asymmetry (11). The public had the extreme information hunger and was easily blinded by information with insufficient veracity, causing more psychological panic. Uncertainty about information quality and misjudgment to the economy made the decision-making more complex, and the investors tended to reduce the difficulty in irrational ways as before (12, 13), which is regarded as emotional irrational judgments (14)). If such sentimental atmosphere occupies dominantly in the stock market, it would intensify the fluctuations and contagion of panic, forming investment behaviors that deviate from ration and triggering volatility. Nonetheless, the exact mechanism between the COVID-19 epidemic and turbulence in the stock market is still under intense academic debate. The investor sentiment performs not only as an inducement of stock market movement and macroeconomic environment but also a direct target consequence by the shakeout.

Undoubtedly, the spread of COVID-19 has affected the level of strategic decision-making and investment behaviors, and the attitude and emotional factors from the investors would, in turn, greatly influence the future stock market performance. The investor sentiment index could behave as the proxy variable to reflect the psychological condition of investors, which would potentially act as a bridge between the impact of COVID-19 and the volatility of stock market during the epidemic. Sergi et al. (15) studied the impact of the increase in Barro Misery Index (BMI), coupled with the percentage of COVID-19 cases adversely on the stock returns and volatility in 76 countries, with different degrees from the developed countries to the emerging markets. Lyócsa et al. (16) found that the fear for COVID-19, manifested as the excess Google search volume activity, signified a timely and valuable data source for forecasting stock price variation. Sun et al. (17) investigated the influences of COVID-19 on Chinese stock market and examined the effect of individual investor sentiment on returns by an event study, showing evidence that the epidemic could cause widespread negative emotions, which led to investor anxiety and market turmoil, and there existed stronger positive correlation between individual investor sentiment and stock returns than usual. Subramaniam and Chakraborty (18) constructed a COVID-19 fear index with the search volume index (SVI) from Google Trends of the search terms to capture the investor mood and highlighted a strong negative association with stock returns that persisted for a significant period without being reversed soon. Xia and Chen (19) observed the stock market performance during the initial outbreak of COVID-19 in relation to Twitter text sentiment analysis, showing strong relationship to COVID-19 sentiment derived from tweets. Chou et al. (20) utilized a deep learning stock market prediction model *via* LSTM, demonstrating that the emotional tendencies and the attention mechanism from investors could help predict closing prices despite the uncertainty of the pandemic, which allows shareholders and investors to understand market forces and emphasizes sustainable investment and development.

In our work, we wish to study when and at what frequencies COVID-19 might synchronously have impact on stock market indexes in both U.S. and China in favor of spatiotemporal interactions over investor sentiment indexes through time series analysis techniques (21–26). To address the potential periodicity, time-frequency co-movement and lead-lag effects in non-stationary time series, wavelet analysis is regarded as a mathematically powerful tool to explain the occurrence of transient events and accommodate to conditions where the amplitude of the response varies excessively for the reason that it could be approximated within a certain time period to a certain frequency band at the same time (24, 27). As the extended usage of wavelet transformation, wavelet coherence and phase difference can be utilized to recognize whether two time series are quantitatively connected by a certain correlation even causality relationship. Matos et al. (28) assessed the conditional relationship in the time-frequency domain between the return on SPX 500 and COVID-19 confirmed cases and deaths, by partial coherencies, phase-difference diagrams, and gains, and found the usefulness of low frequency cycles of U.S. stock market index in anticipating the cycles of deaths in an anti-phasic way. Su et al. (2019) utilized continuous wavelet analysis and aimed to assess whether the causality of geopolitical risk, oil prices, and financial liquidity supported the monetary equilibrium model in Saudi Arabia. Sharif et al. (29) revealed the unprecedented impact of COVID-19 and oil price shocked on geopolitical risk levels, economic policy uncertainty, and low-frequency band stock market volatility with the coherent wavelet and coherent wavelet-based Granger causality tests. Goodell and Goutte (30) addressed the stock market evidence and employed econometric procedures, including wavelet coherence and neural network analyses, to rigorously examine the role of COVID-19 and found co-movements between cryptocurrencies and equity indices. Jiang and Yoon (31) studied the dynamic co-movement between oil and six stock markets with wavelet multi-scale decomposition and coherence, and discovered the feedback relationships by maximal overlap discrete wavelet transformation. Amar and Carlotti (32) adopted multivariate continuous wavelet to investigate the strength and the magnitude of the relationship between the economic policy uncertainty index and the implied volatility index, indicating more persistent intensity of the interdependence between the stock markets and uncertainty indices in the crisis and risk periods. Ghosh et al. (33) analyzed the inherent evolutionary dynamics of financial and energy markets by observing their interrelationships with the continuous wavelet transformation, and suggested the existence of a strong trend component and long-range dependence.

In this paper, we quantitatively investigate the time-frequency co-movement impact of COVID-19 on U.S. and China stock market since early 2020 in terms of daily observation from NDX, DJIA, SPX, SSEC, SZI, and propose to explore the divisibility and the predictability to the volatility of stock market during the development of COVID-19. The proliferation of the virus has led the investors into great uncertainty, thus inducing a series of negative emotions and anxiety, influencing their investment judgment. Amid this situation, this paper focuses on the following specific questions. First, to what extent has the growth in the number of confirmed COVID-19 diagnoses contributed to the volatility of U.S. and Chinastock markets during the pandemic outbreak? In comparison, would U.S. and Chinastock markets be equally sensitive to COVID-19 impact? Second, if COVID-19 could be regarded as one cause of stock index fluctuations, how much does it correlate with emotion and psychological panic of investors? Are sentiments of investors within the same level between China and U.S.? Third, is there any variability and regularity about the COVID-19 impact on the stock markets at different stages in such a black swan event? Have the reactions of the investors to the stock markets varied over time? Fourth, to what extent do the economic policies that materialized amid the pandemic have an attribution on easing the psychological expectations of investors about stock markets? Has this impact likewise changed over time?

The remainder of the paper is organized as follows: section Materials and Methods describes the access of multivariate time series referred and outlines the basics in wavelet coherence and phase difference in our study. section Results quantitatively and systematically investigates the time-frequency co-movement in stock market indexes regarding newly confirmed number of COVID-19 cases per day. Section Discussion suggests potentially mutual relationships between COVID-19 impact and stock market indexes along the time domain, together with the investor sentiment indexes. Finally, the conclusion is drawn in section Conclusion.

## Materials and Methods

### Data

#### COVID-19 Statistics

During the COVID-19 epidemic, a large amount of daily statistics springs out online, e.g., from WHO, COVID-19 Public Dataset of Google Cloud, Johns Hopkins Center, including the number of newly confirmed cases, deaths, and suspected cases per day. In this study, we retrieved the daily number of confirmed COVID-19 cases for weekdays in the U.S. and China, respectively, from JHU and the National Health and Wellness Commission of P. R. China, and then made the preprocessing with the natural logarithm.

#### U.S. Stock Index

We applied the three major New York stock indexes, the benchmarks of the U.S., and global stock markets, i.e., the National Association of Securities Dealers Automated Quotations Index (NDX), Dow Jones Industrial Average (DJIA), S&P 500 Index (SPX), from January 1, 2020 to April 31, 2021, to investigate the impact of COVID-19 on U.S. stock market performance. The daily stock closing price of the three indexes is available from Yahoo Finance, and we also first made the natural logarithm.

#### China Stock Index

We accessed Shanghai Stock Exchange Composite Index (SSEC) and Shenzhen Composite Index (SZI) from the official tracker to portray the Chinese stock market performance, which constitutes the most fundamental and essential references for Chinese investors and securities practitioners and institutions, and made the preprocessing with the natural logarithm.

#### U.S. Investor Sentiment Index

We utilized Sentix Investor Confidence from Sentix Global Investor Survey for U.S. in our study, which collects the weekly estimation of 14 financial markets from, currently, more than 5,000 private and institutional investors. When the majority of the survey respondents are optimistic about the economy, the sentiment index value is >0, conversely; the value tends to be negative when the investor sentiment is pessimistic.

#### Chinese Investor Sentiment Index

We made use of Chinese investor sentiment index from China Securities Investor Protection Fund Limited Liability Company to investigate the changes in the psychological expectations of Chinese investors during pandemic. When the majority of the survey respondents are optimistic about the economy, the sentiment index value is >0, conversely; the value tends to be negative when the investor sentiment is pessimistic. The value of Chinese investor sentiment index ranges from 0 to 100, with 50 being a neutral value. When the proportion of investors with optimistic views is greater than that with pessimistic views, and the overall investor confidence is expected to be optimistic, the index value is >50, otherwise, lower than 50. Since the definition of Chinese investor sentiment index is highly similar to the U.S. investor sentiment index, we made the normalization and assumed to be highly comparable for each other.

### Methods

#### Basic Wavelet Analysis

The basic wavelet analysis is to make its decomposition of the daily number of COVID-19 infections *X*(*t*) into a superposition of multiple wavelet functions; all of which are derived from a parent wavelet function, with the scaling and translation that localize in both frequency and time domain, defined as follows:


(1) $$\begin{array}{l} \phi_{a,\tau }=\frac{1}{\sqrt{a}}\phi \left(\frac{t-\tau }{a}\right)\left(a,\tau \in R,a>0\right) \end{array}$$

(2) $$\text{with}\int_{-\infty }^{+\infty }\phi (\text{t})\text{dt}=0$$

where ϕ_*a*,τ_(*t*) is a sub-wavelet, with ${\left||\phi_{a,\tau }|\right|}^{2}=1$, *a* is a scaling parameter, ensuring the compressed or stretched variance of the wavelet function, and τ is a shift parameter about the deviation location of the wavelet function, ϕ(*t*) is a mother wavelet function, ϕ(*t*)ε*L*^2^ (*R*), which could take a wide variety of forms, such as Haar wavelet (34), Morlet wavelet (35), Daubechies wavelet (36), Meyer wavelet (37), Mexican straw hat wavelet (38) and so on. Thus, the wavelet analysis is able to detect higher or lower-frequency components of the examined COVID-19 time series *X*(*t*) characterized with the irregularity and non-stationarity along the time domain.

Taking into consideration of the daily increase of COVID-19 confirmed cases *X*(*t*) at multiple timescales, we try to adopt the principle of continuous wavelet transformation over time:


(3) $$\begin{array}{l} W_{X}\left(a,\tau \right)=\frac{1}{\sqrt{a}}\underset{R}{\int }X\left(x\right)\bar{\phi }\left(\frac{t-\tau }{a}\right)dt \end{array}$$

where *W*_*X*_(*a*, τ) is the wavelet transform coefficient.

#### Wavelet Coherence

Let *S*(*t*) be the corresponding closing price of the stock market index. The cross-wavelet transform between the daily number of new confirmed COVID-19 cases *X*(*t*) and the daily stock closing price *S*(*t*) can be expressed as:


(4) $$\begin{array}{l} W_{XS}\left(a,\tau \right)=W_{X}\left(a,\tau \right)W_{S}^{*}\left(a,\tau \right) \end{array}$$

where *W*_*X*_(*a*, τ) and *W*_*S*_(*a*, τ) are, respectively, the continuous wavelet transforms of *X*(*t*) and *S*(*t*), and $W_{S}^{*}\left(a,\tau \right)$ represents the complex conjugation of*W*_*S*_(*a*, τ). The local covariance between the cross-wavelet power spectrum is then defined as follows:


(5) $$\begin{array}{l} |W_{XS}\left(a,\tau \right)|=\sqrt{\left|W_{X}\left(a,\tau \right)||W_{S}^{*}\left(a,\tau \right)\right|} \end{array}$$

Since the cross-wavelet transform emphasizes the distribution with the high common power, the cross-wavelet power spectrum |*W*_*XS*_(*a*, τ)| thus measures the mutual local covariance at each frequency and each time scale, indicating where the time series have high common power in the time-frequency domain. Suppose that there exists the potential periodicity of the time series, we attempt to extend the finite length to limit the edge effects. The cone of influence (COI) designates the regions in the scalogram potentially affected by the edge-effect artifacts. When the wavelet power spectrum amplitude decreases, there will be more discontinuities in edges, which is subject to boundary distortion, and hereby provides unreliable reference that should be removed.

The correlation between the daily increase of COVID-19 cases and the stock market index for each country can be further identified by wavelet coherence, which helps ascertain the specific region with co-movement patterns in the time-frequency domain, as follows:


(6) $$\begin{array}{l} C_{XS}\left(a,\tau \right)=\frac{\left|A\left(W_{XS}\left(a,\tau \right)\right)\right|}{\sqrt{A\left({\left|W_{XX}\left(a,\tau \right)\right|}^{2}\right)\cdot A\left({\left|W_{SS}\left(a,\tau \right)\right|}^{2}\right)}} \end{array}$$

where *A*(*W*_*XS*_(*a*, τ)) is the cross spectral density between *X*(*t*) and *S*(*t*), 0 ≤ *C*_*XS*_(*a*, τ) ≤ 1. The wavelet coherence coefficient allows to detect the synchronization similarity. The higher wavelet coherence is the stronger dependence between two time series. The lower value of wavelet coherence indicates the possible absence of correlation, and *vice versa*.

Meanwhile, the wavelet phase differences dedicate to distinguish either the negative or positive correlative directions, evaluating the possible delay in the oscillation, or the lead-lag effects between COVID-19 and stock market indexes:


(7) $$\begin{array}{l} \psi_{XS}=\tan^{-1}\left(\frac{I\left\{A\left(W_{XS}\left(a,\tau \right)\right)\right\}}{R\left\{A\left(W_{XS}\left(a,\tau \right)\right)\right\}}\right),\psi_{XS}\varepsilon \left[-\pi ,\pi \right] \end{array}$$

where *I* and *R* are the imaginary and real parts of the smoothed cross-wavelet transform, respectively. If ψ_*XS*_ is zero, the two-time series co-move or link together in-phase at the specific frequency, and if it is π (or −π), they co-move in opposite directions out of phase. If ψ_*XS*_ε(0, π/2), *X*(*t*) leads *S*(*t*) and it shows positively co-moves; if ψ_*XS*_ε(π/2π), *S*(*t*) leads *X*(*t*) and it shows negatively co-moves; if ψ_*XS*_ε(π, π/2), *X*(*t*) leads *S*(*t*) and it shows negatively co-moves; if ψ_*XS*_ε(−π/2, 0), *S*(*t*) leads *X*(*t*) and it shows positively co-moves.

### Results

**Figure 1:**
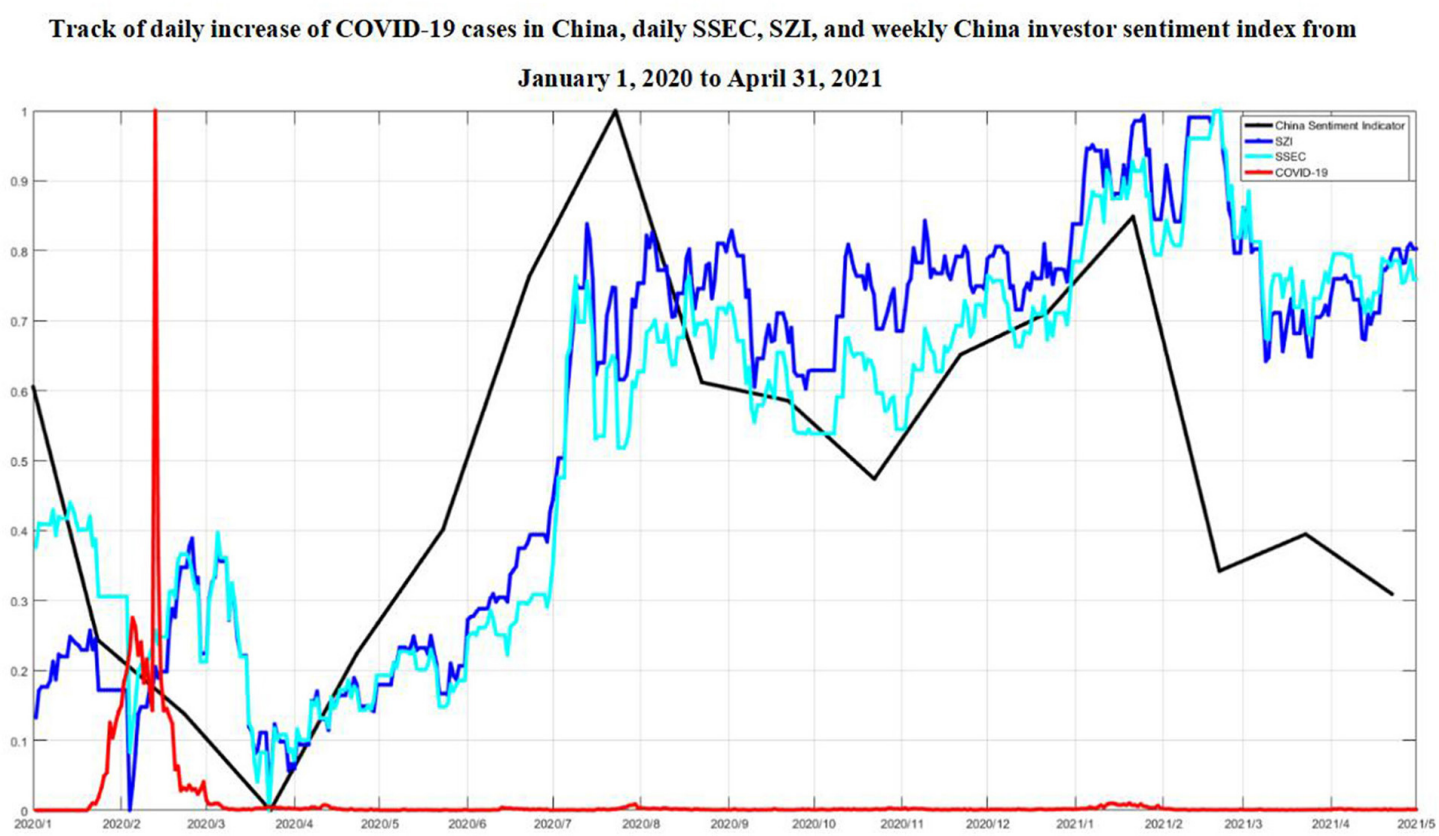
Track of daily increase of COVID-19 cases in China, daily SSEC, SZI, and weekly normalized China investor sentiment index from January 1, 2020 to April 31, 2021.

**Figure 2:**
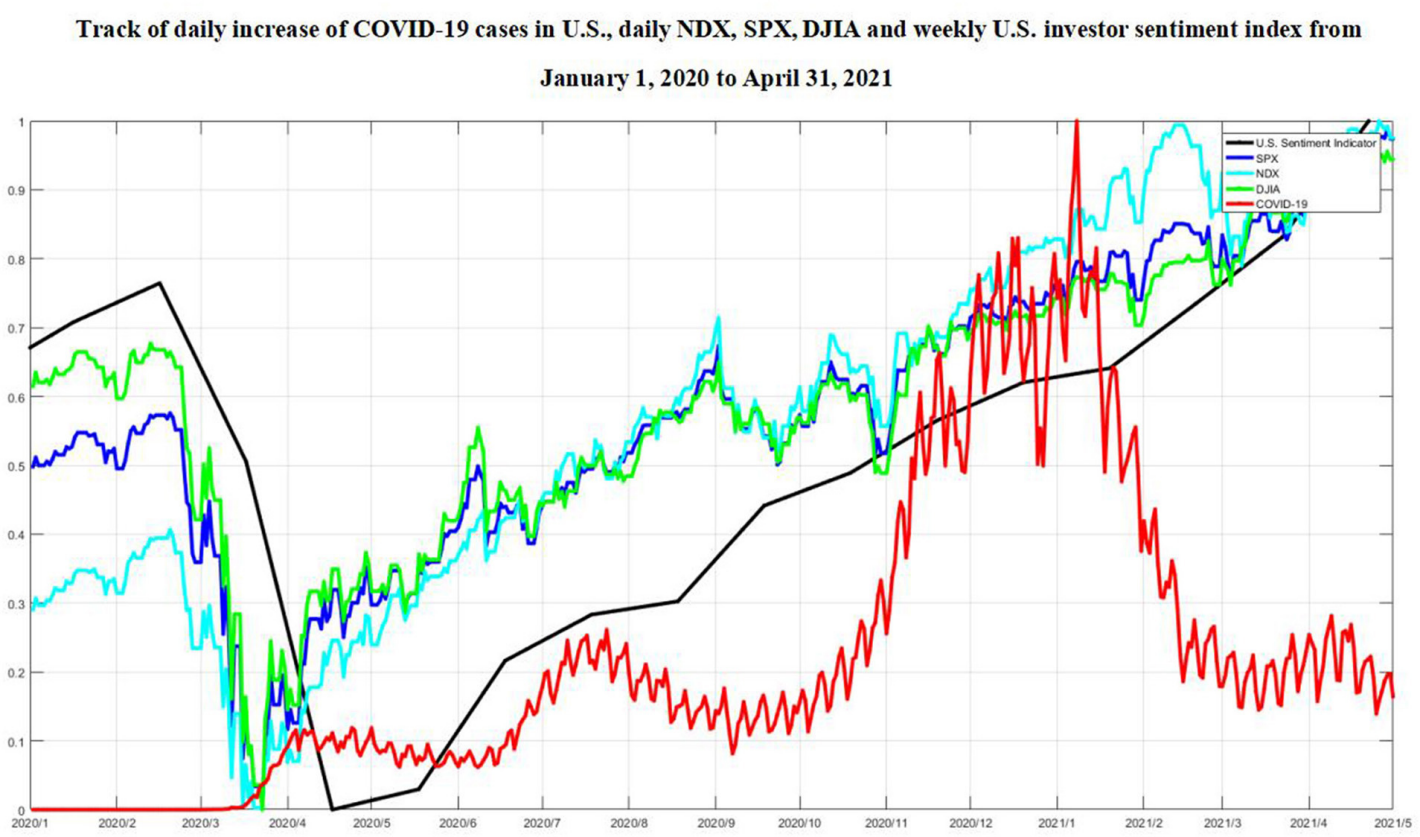
Track of daily increase of COVID-19 cases in U.S., daily NDX, SPX, DJIA, and weekly normalized U.S. investor sentiment index from January 1, 2020 to April 31, 2021.

The track of the raw time series about the number of new confirmed COVID-19 cases per day, the daily stock market indexes, and the weekly normalized investor sentiment indexes for both China and U.S. from January 1, 2020 to April 31, 2021 has been listed in Figures 1, 2.

We utilized the wavelet coherence to search for when and at what frequencies and directions the daily stock market indexes would synchronously move together with the number of new confirmed COVID-19 cases per day from January 1, 2020 to April 31, 2021, and described the possible lead-lag effects through the wavelet phase difference.

**Figure 3:**
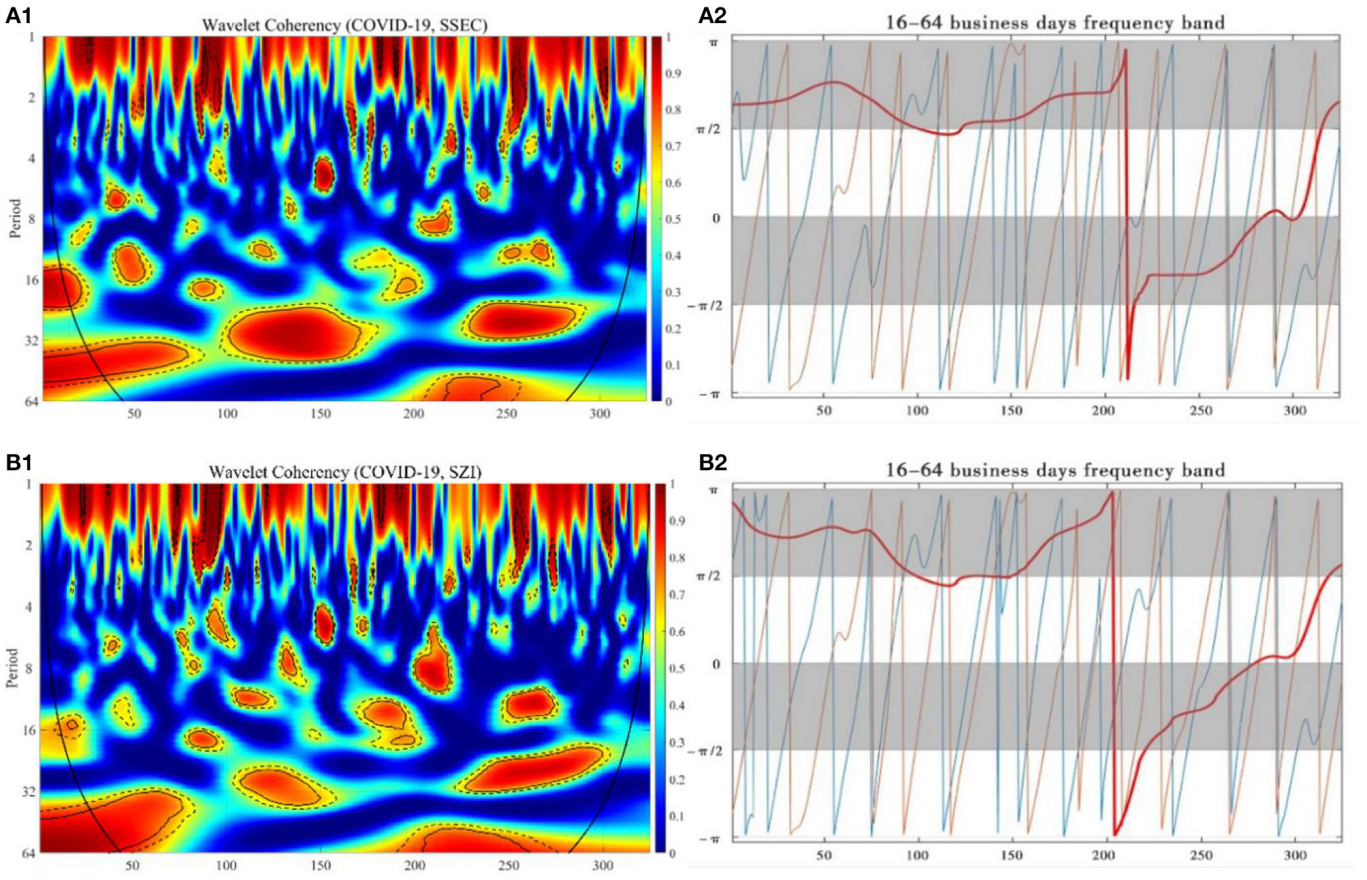
The wavelet coherence and phase difference between daily COVID-19 new cases in China and SSEC, SZI. Wavelet coherence (**A1,B1**) and phase difference (**A2,B2**).

**Figure 4:**
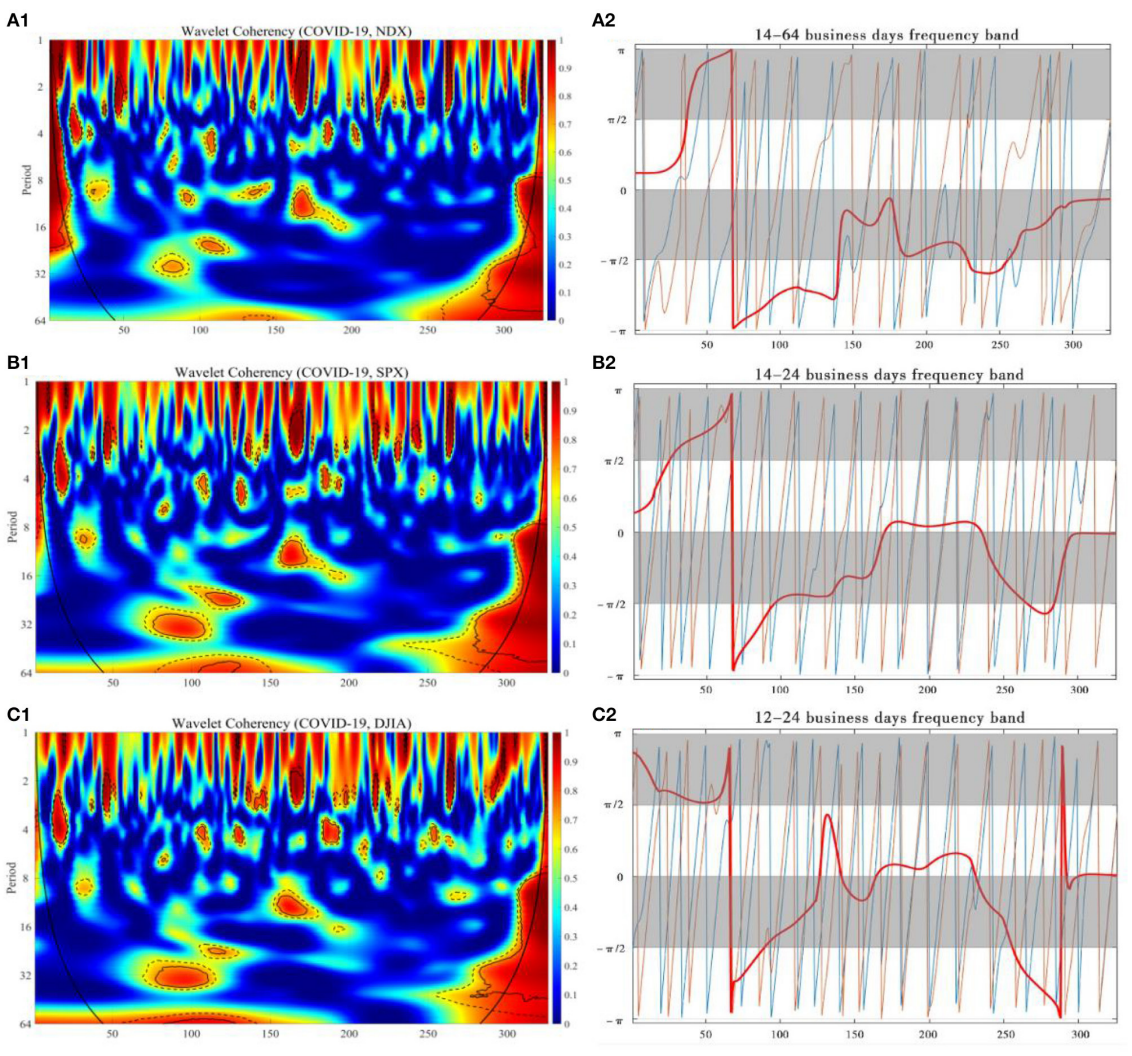
The wavelet coherence and phase difference between daily COVID-19 new cases in U.S. and NDX, SPX, DJIA. Wavelet coherence **(A.1, B.1, C.1)** and phase difference **(A.2, B.2,C.2)**.

The wavelet coherence and phase difference map between the daily increase of COVID-19 cases in each country and SSEC, SZI, DJIA, SPX, and NDX have been calculated and, respectively, shown in Figures 3, 4, where the y-axis refers to frequency in business days, and the x-axis refers to the time domain. The partial wavelet correlation has been characterized by its discontinuity, corresponding to the relative intensity at each frequency in aid of the color bar representation, where the colors range from weaker coherency in blue to stronger coherency in red. The cone of the coherence map, called COI (the cone of influence) margin, is indicated in the black convex curve, with 5 and 10% significance levels, respectively, represented by thin black lines and dashed black lines. Regarding all kinds of possibilities in terms of partial phase difference involving SSEC, SZI, DJIA, SPX, and NDX and daily new confirmed cases of COVID-19 in each location, a higher incidence of regions with strong partial coherency is plotted here.

For COVID-19 vs. SSEC, in Figures 3A.1,A.2, the correlation coefficient in the 15–25 business days band is >0.95 during 1–25 business days from January 1, 2020 to February 12, 2020 and 40-60 business days from March 4, 2020 to March 31, 2020. The correlation coefficient in the 20–35 business days band is >0.95 during 100–170 business days from June 2, 2020 to September 10, 2020 and during 225–280 business days from December 7, 2020 to February 26, 2021 and the correlation coefficient in the 32–60 business days band during 1–76 business days between January 1, 2020 and April 24, 2020 and during 220–248 business days from November 27, 2020 to January 8, 2021, indicating that there exists a significant long-term correlation. Meanwhile, in the long-term frequency band, the phase difference for both variables is in the range of π to π/2 throughout the period when the correlation is strong, which implies that there is significantly negatively correlated between the two time series.

For COVID-19 vs. SZI, in Figures 3B.1,B.2, with reference to the wavelet coherence map, we found it exceeded 0.95 in the 55–64 business days band during 1–75 business days from January 1, 2020 to April 23, 2020 and during 205–255 business days from November 9, 2020 to January 9, 2021 in a long-term perspective, and it exceeded 0.85 in the 28–35 business days band during 100–150 business days from June 2, 2020 to August 13, 2021 and exceeded 0.95 in the 25–35 business days band during 230–300 business days from December 11, 2020 to March 26, 2021, indicating significant correlation in the midterm. The phase difference implies that, in the long-term frequency band, the two times series are significantly negatively correlated throughout the time period.

For COVID-19 vs. NDX, in Figures 4A.1,A.2, the correlation coefficient in the short-term frequency band of 4–8 business days was about 0.95 from 1 to 20 business days from January 1, 2020 to February 5, 2020, but, after that, it turned to a weak correlation. The midterm band of 10-15 business days appears during 155-175 business days from August 20, 2020 to September 17, 2020. In particular, after 280 business days from the beginning, that is February 26, 2021, the correlation in the long-term band of 8–32 business days increases significantly, and this correlation continues until 325 business days, that is April 30, 2021, during which the correlation coefficient reaches about 0.95. The phase difference demonstrates that there are initially negative correlations between two time series for both variables at the very beginning, and then the correlation turned out to be positive significantly during the entire co-movement period, and the COVID-19 tends to lead its positive changes in NDX.

For COVID-19 vs. SPX, in Figures 4B.1,B.2, the short-term correlations in the 2–4 business days band emerge during 1–10 business days from January 1, 2020 to January 14, 2020 and during 20–30 business days from February 5, 2020 to February 20, 2020, starting from the first business day. The mid-term band of 10-15 business days during 155–175 business days from August 20, 2020 to September 17, 2020. Long-term correlations of 20–35 business-day bands appear in the 70–110 business day range from April 16, 2020 to June 16, 2020 and 110–135 business day range from June 16, 2020 to July 23, 2020. Starting from 280th business day in our time span, which is March 11, 2021, a high correlation emerges for the 8–32 business day band with a correlation coefficient around 0.95, and such correlations last for a range of 280–350 business days from March 11, 2021 to April 30, 2021. The phase difference showed an almost significant positive correlation between the two time series of the two variables during the most isotropic motion with a short period of negative correlation at the beginning time, and that the new coronary pneumonia tended to dominate the changes in its SPX.

For COVID-19 vs. DJIA, in Figures 4C.1,C.2, the correlation coefficient in the long-term frequency band of 28–35 business days was about 0.9 during 70–120 business days from April 16, 2020 to July 2, 2020, but, after that, it turned to a weak correlation. The mid-term band of 10-15 business days appears during 150-180 business days from August 13, 2020 to September 24, 2020. It is worth mentioning that the correlation in the long-term band of 8–32 business days is significantly stronger, starting during 290–350 business days from March 11, 2021 to April 30, 2021 during 275–350 business days from February 19, 2021 to April 30, 2021, with a correlation coefficient of about 0.95, and the long-term band of 55–64 business days during 80–125 business days from April 30, 2020 to July 9, 2020 during 100–125 business days from June 2, 2020 to July 9, 2020. The phase difference demonstrates that there is significantly positive correlation between two time series for both variables during the most co-movement period with a short time of negative correlation at the beginning and the end part of the time span, and the COVID-19 tends to dominantly lead its changes in DJIA.

## Discussion

First, it has been basically revealed from our experiment results that, in China, both SSEC and SZI indexes synchronously shared dominantly negative correlative effects with the increased number of COVID-19 cases in a long-term band, including the 30th-75th, 110th-150th, 220th-280th business days since January 1, 2020, i.e., from February 19 to April 23, 2020, from June 16 to August 13, 2020, from November 30, 2020 to February 25, 2021. The increase of COVID-19 confirmed cases exerted dramatic negative influences to both the SSEC and SZI indexes from the perspective of midterm frequency as well, especially for SSEC at the very beginning of the pandemic during the 1st-40th business days, from January 1 to February 12, 2020.

As the first hardest-hit country of COVID-19 pandemic, China took the immediate and unprecedented actions in public health history to prevent the further outbreak of the epidemic, and the first epicenter, the city Wuhan, has been completely locked down since January 23, 2020. It is widely known that, 17 years ago, severe acute respiratory syndrome (SARS), another emerging coronavirus, initially attacked mainland China and led to a total of 5,327 confirmed cases with 343 deaths (39). Such experiences in defeating coronavirus with quarantining were still fresh in memory of most Chinese investors, and hereby possibly resulted in strong panic and anxiety, which would speed up the value of the investor sentiment index into the pessimistic tendency toward the stock market.

Afterward, WHO announced COVID-19 to be classified as a public health emergency of international concern. The macroeconomic environment has experienced a high correlation with the impact of the COVID-19 pandemic during that time period and radiated into the financial market volatility. On the first trading day after Chinese New Year, i.e., February 3, 2020, SSEC dived 7.72% to close at 2746.61 points, and SZI fell 8.45% to close at 9,779.67 points as stock markets reopened, remaining a low level of consolidation with a total of 3,188 stocks falling in both China stock markets, and the combined daily market return below −9%, which brought the biggest drop since 2015, and Shanghai index intraday hit the largest decline since 23 years. Shanghai Composite main net capital outflow was 14.44 billion yuan, Shenzhen Composite net capital outflow was 20.69 billion yuan, with Shanghai Stock Exchange net capital inflow of 1.92 billion yuan, and Shenzhen Stock Exchange net capital inflow was 4.39 billion yuan. The normalized Chinese investor sentiment index fell all the way to 0.2 during this week, compared with 0.5 in the first week of 2020.

In the meantime, the increase of the irrational judgment toward COVID-19 has greatly influenced the behaviors of investors, leading to the possible overreaction in China stock market. One of the specific manifestations is that the uncertain panic sentiment during the epidemic made most Chinese investors choose to hedge the bets, and the liquidity demand rose. The lack of awareness of the transmission route during the initial stage, coupled with the newness, contagiousness, and the estimated high death rate, triggered public sentiment fluctuations, which, in turn, brought excessively pessimistic expectations, resulting in corresponding volatility in stock markets. To maintain a reasonable abundance of banking system liquidity and stability of the money market during the special period of the epidemic on February 3, 2020, the People's Bank of China carried out 1.2 trillion yuan in reverse repo operations to ensure adequate supply of liquidity, and the overall liquidity of the banking system was 900 billion yuan ahead of the same period last year.

Later on, China took a series of grid governance measures on COVID-19 epidemic prevention and control, including the strict household survey and resident quarantine, city lockdown, etc., and has made significant progress since March, 2020. Confidence of the majority of the investors in China stock market was gradually rebounded from late March to early April, and the sentiment index of the investors tended to reflect their hopes for the sustained economic recovery and the belief in the ability to crush any resurgence of the virus. The average value of normalized Chinese investor sentiment index in the last week of April returned to 0.2. The degree of stock market dynamism had a positive correlation with COVID-19 control. A series of interest rate-cutting policies in major economies around the world had also lowered the benchmark bank interest rates and the expected returns, making stocks more attractive as capital that began to favor higher-yielding assets [(1), Liu et al., 2020].

Since China was among the first countries to quickly resume production and the fiscal and monetary policies collaborated to constitute a joint support to economy, China stock market began to behave quite actively. On July 3, 2020, SSEC went up to 2.01% to close at 3,152.81 points, hitting a historical new high since April 2019, and SZI gained 1.33% up to close at 12433.26 points. Both stock markets soared in heavy trading volume, with turnover breaking a trillion mark in 2 consecutive days, and more than 80 individual shares reached the daily limit up. On July 7, SSEC raised up 0.37% to close at 3,345 points, with a volume of 793.6 billion, the highest daily volume since the end of the last bull market; SZI rose up 1.72% to close at 13,163.98 points. As of July 8, blue-chip Shanghai-Shenzhen 300 Index of China stock market has risen up for 7 consecutive trading days. Within these 7 days, SSEC successively reached 3,000; 3,100; 3,200; 3,300; and 3,400 points, peaking a high record in 5 years. The broader context of the surge was the better prospects, which had significantly boosted market confidence against the backdrop of economy recovery, and the investors combined with highly optimistic expectations and tended to go long on the stock markets with bullish actions. The normalized Chinese investor sentiment index in the first and second weeks of July reached, respectively, 0.8 and 0.9.

As COVID-19 vaccine development advanced, the ease of the epidemic made the socioeconomic environment more stabilized. As of October, 2020, four inactivated COVID-19 vaccine candidates have entered the Phase III clinical trials. China was leading COVID-19 vaccine development, expected to produce up to 610 million doses by the end of 2020. On December 31, 2020, China announced that it had granted conditional marketing authorization for the first COVID-19 vaccine. The normalized Chinese investor sentiment index in the first week of 2021 kept 0.8. The joint prevention and control mechanism against COVID-19 have allowed economy recovery. Although there was still COVID-19 recurrence of small scale that led to localized blockades from late 2020 to early 2021, the stock market showed no significant sign of being slowed down, and the corresponding drop in investor sentiment index no longer displayed the decline dramatically, only varied around.1. Chinese equities proved to be one of the most resilient stock markets and were backed by the steady work resumption and stimulus policies under COVID-19 development (40).

Second, it has been demonstrated that a surprisingly strong positive correlation between the increased number of COVID-19 cases and NDX, DJIA, and SPX from our experiment results in a long-term band, around the 80th–125th and 160th–175th after 290th business day since January 1, 2020, i.e., from April 30 to July 9, 2020, from August 27 to September 17, 2020 after March 11, 2021. As U.S. started to suffer from the epidemic, a brief and sharp negative correlation has been obviously observed at the very beginning, and U.S. stock market has experienced consecutive trading halts due to coronavirus concerns.

The first stock market crash began on March 9, 2020, with the largest point plunge of 3.37% for DJIA up to that date in history, 7.21 and 5.88% drops for NDX and SPX, followed by more record-setting point drops within the next 2 weeks. On March 12, U.S. stock market plunged 7.2% in <6 min after the opening bell, triggering a circuit breaker that halted trading, and DJIA closed at 23,553.22 after declining 5.9%, directly breaking the 2008 record for the largest one-day drop since the first U.S. stock temporarily halted trading in 1987, and the broader SPX reached 2,741.38 after decreasing 4.9%, and the tech-laden NDX hit 7,952.05 after losing 4.7%. On March 14, 2020, U.S. declared to struggle with the outbreak of the COVID-19 pandemic by launching a $50 billion reserve of emergency funds for state health care agencies, which has been viewed as a favorable signal on Wall Street and most investors, sending DJIA up to 1,985 points, or 9.4%, its best gain since October 2008. On March 15, the Federal Reserve System decided to lower the target range for the federal funds rate to 0–0.25%, and a massive $700 billion quantitative easing program was also announced, including the repurchase of at least $500 billion holdings in Treasury securities and the addition of at least $200 billion holdings in agency mortgage-backed securities. On March 16, worries about COVID-19 and stock market performance still sent DJIA plunging 2,997 points, or 12.9%, and SPX futures dropped 12%, triggering a circuit breaker, and NDX closed down −12.32% since its worst day on October 19, 1987. To counter the impact of COVID-19, on March 17, U.S. government announced to launch a $1 trillion stimulus package that includes tax cuts, cash checks totaling $500 billion, and the initiation of the Federal Reserve to the monetary policy tools helped make up economic losses due to the epidemic spreading across the U.S. On March 18, the U.S. stock market triggered the circuit breakers again for the fifth time as DJIA jumped 5.2% or 1,048.79 points to close at 21,237.31, with NDX closed at 7,334.78, soaring 6.2% or 430.19 points, and SPX climbed 6% or 143.06 points to close at 2,529.19. It is assumed from our experiment results that at the very beginning of COVID-19 outbreak in U.S., the crash of stock market reflected the worries of the investors about the socioeconomic impact of the pandemic, and there was a short-term negative attitude toward the impediment of the epidemic to economic development, with short-term bearishness predominating and thus causing strong volatility in the financial markets (16). Their uncertainty about the danger of the coronavirus and the high transmission rate, plus the shutdown of businesses and industries, the possibility to be laid off with a high unemployment rate, the decreased purchasing power, all caused a great deal of fear and insecurity among investors, made them lose the confidence in stock market, and resulted in the value of the investor sentiment index toward the pessimistic tendency. The normalized U.S. investor sentiment index dropped sharply from early March and reached its minimum in late April, during the entire time domain of our study, compared with 0.7 in the first week of 2020.

Although COVID-19 spread constituted one major driving force behind the unprecedented stock market crash, it did not last long. The stock market experienced surprising recovery, and investor confidence came back quite soon, propelled by a combination of federal stimulus and vaccine development for the pandemic, with the normalized U.S. investor sentiment index around 0.4 averagely from late August to late September. The sharp turnaround of the stock market has, nonetheless, created the most uneven recovery in the U.S. history, according to the Washington Post. On August 26, 2020, DJIA gave up 60.02 points, or 0.2%, to close at 28,248.44, with SPX added 12.34 points, or 0.4% to close at 3,443.62, and NDX closed at 11,466.47, adding 86.75 points, or 0.8%, which all set a new recorded high point. Despite the rockiness of September, Wall Street bagged its best back-to-back quarters since 2009, with SPX growing 8.5% and DJIA gaining 7.6% in the third quarter. The National Association of Securities Dealers Automated Quotations Index added 11% from July through September for its best two consecutive quarters since early 2000s.

On November 9, 2020, Pfizer announced their mRNA-based vaccine candidate against COVID-19, jointly developed with BioNTech, achieved success in first interim efficacy analysis from Phase 3 clinical study, which demonstrated evidence over 90% effectiveness in preventing COVID-19 among the participants without prior SARS-CoV-2 infection. Pfizer shares hereby rose as high as $41.99 that day and closed up to 7.69% at $39.20. DJIA gained 3% or 834.57 points to end the day at 29,157.97, marking its best day in terms of percentage gains since June 5, with SPX increased 1.17% or 41.06 points to close at 3,550.50; the tech-heavy NDX declined 1.5% or 181.45 points to end at 11,713.78. By December 31, 2020, DJIA grew up 64.6% from its low point on March 23 in the pandemic outbreak, with SPX and NDX up a whopping 67.9 and 87.9%, respectively, from their March lows. The global equity asset binge was directly related to the mega-positive news coming out of Pfizer vaccine development, with investors being quite optimistic about the expectation in stock market, and the normalized U.S. investor sentiment index got to 0.6 in late 2020.

The American Rescue Plan Act of 2021, a $1.9 trillion COVID-19 relief package, was signed into law on March 11. On March 31, 2021, DJIA slid 0.3% or 104.41 points to close at 33,066.96 from a record closing high in the previous session, which has risen 65.7% from the low point of the epidemic eruption last March; SPX fell 0.3% or 12.54 points to end at 3,958.55 points, while the tech-heavy NDX declined.1% or 14.25 points to finish at 13,045.39 points. In the first quarter of 2021, major equity indexes reached record highs; SPX and NDX rose 93.1 and 77.6%, respectively, from their March lows, 2020. Several benchmark stock market indexes, DJIA, SPX, and NDX, posted noteworthy gains. NDX underperformed slightly because of higher bond yields, which adversely affected the valuation of technology companies that have high price-to-earnings ratios. The primary reason for the higher DJIA was the epidemic easing and economic recovery signals received by the investors, gradually revitalizing the dollar and, therefore, allowing for greater liquidity. During the spread of COVID-19, multiple economy stimulus bills and measures from the U.S. government, including cash payments to taxpayers, increase in unemployment insurance and rental assistance, further encouraged investor sentiment, leading to additional gains in the stock market. The normalized U.S. investor sentiment index raised from 0.6 in January to 0.8 in March, indicating the steady positive expectation among investors.

In general, in view of spatiotemporal co-movement impact of COVID-19 pandemic since 2020, we conclude the external responses of both China and U.S. stock market indexes could be roughly divided into three distinguished phases in a long-term band, synchronized with the investor sentiment indexes, namely, the time period of 30–75, 110–150, and 220–280 business days for China, and the time period of 80–125 and 160–175 after 290 business days for U.S. At the first phase, the reason for the dramatic decline in both China and U.S. stock market indexes can be mainly attributed to the sudden attack and rapid rise of COVID-19 confirmed cases due to the uncertainty of the danger of the coronavirus and the high transmission rate, and the pessimistic economic expectations from the investors in both countries. In addition, although both China and U.S. stock market indexes similarly made a sensitive reaction to COVID-19 impact, and there were strongly shared negative correlations from the beginning of the epidemic in both countries, the significant COVID-19 impact on Chinese economy has been recognized roughly 50 business days earlier than the U.S. market; the rapid outbreak of the emerging coronavirus, plus the extreme horror of the past SARS experiences, has been highlighted into the downward trend of the Chinese stock market, and, at that time, the effect on US stock market could even hardly be observed. Later on, U.S. experienced unprecedented succession of a trading halt, with the impact of the COVID-19 sending stock indexes a cliff fall. The extreme volatility in both stock markets was more correlated with its negative returns, and the epidemic was more likely to further exacerbate market panic, consistent with the leverage effect (41, 42), exhibiting the strong asymmetries. At the second phase, thanks to a series of COVID-19 prevention measures globally taken in response to the black swan event, the revitalization of China and U.S. stock markets shared strong simultaneous moves but interestingly exhibited quite opposite responses to the COVID-19 impact. Although the Chinese stock market still reacted ahead, with regard to the variation in COVID-19 development, compared with the U.S. stock market, the former tended to receive optimistic feedback from the majority of investors until China made a great success in controlling and preventing the spread of virus; on the contrary, the latter showed the intensive confidence recovery from the investors even if the daily cumulative increase of COVID-19 continued to rise. Therefore, China stock market made a boost after the substantial control to COVID-19 cases, revealing there was still a consistently significant negative correlation between stock market indexes and the increase of COVID-19 cases, while the U.S. stock market behaved surprisingly less sensitive with regard to the rapid growth of new confirmed COVID-19 cases, yielding the economy revitalization positively correlated with COVID-19 impact throughout the period, indicating the differences in investor sentiment from the two countries, with the former relatively conscientious and the latter more aggressive under the development of COVID-19. At the third phase, the great progress in COVID-19 vaccine development, as well as the sustainable economic stimulus, began to impose both direct and indirect forces to the stock market in both countries, especially for U.S., which gradually stepped into a relatively orderly control to the COVID-19 epidemic. In the meantime, China kept on the iron fist measures to govern the virus outbreak, and only a few occasional COVID-19 cases occurred during the time period, which benefitted a lot to promote the performance growth in the stock market. The vulnerability to COVID-19 diminished to some extent as the uncertainty in the minds of the investors gradually decreased, and the investor sentiment index values rebounded with the insensitive tendency. The slowly growing number of confirmed cases in both countries corresponded to the negative correlation with the strengthening increase of stock market performances.

**Figure 5:**
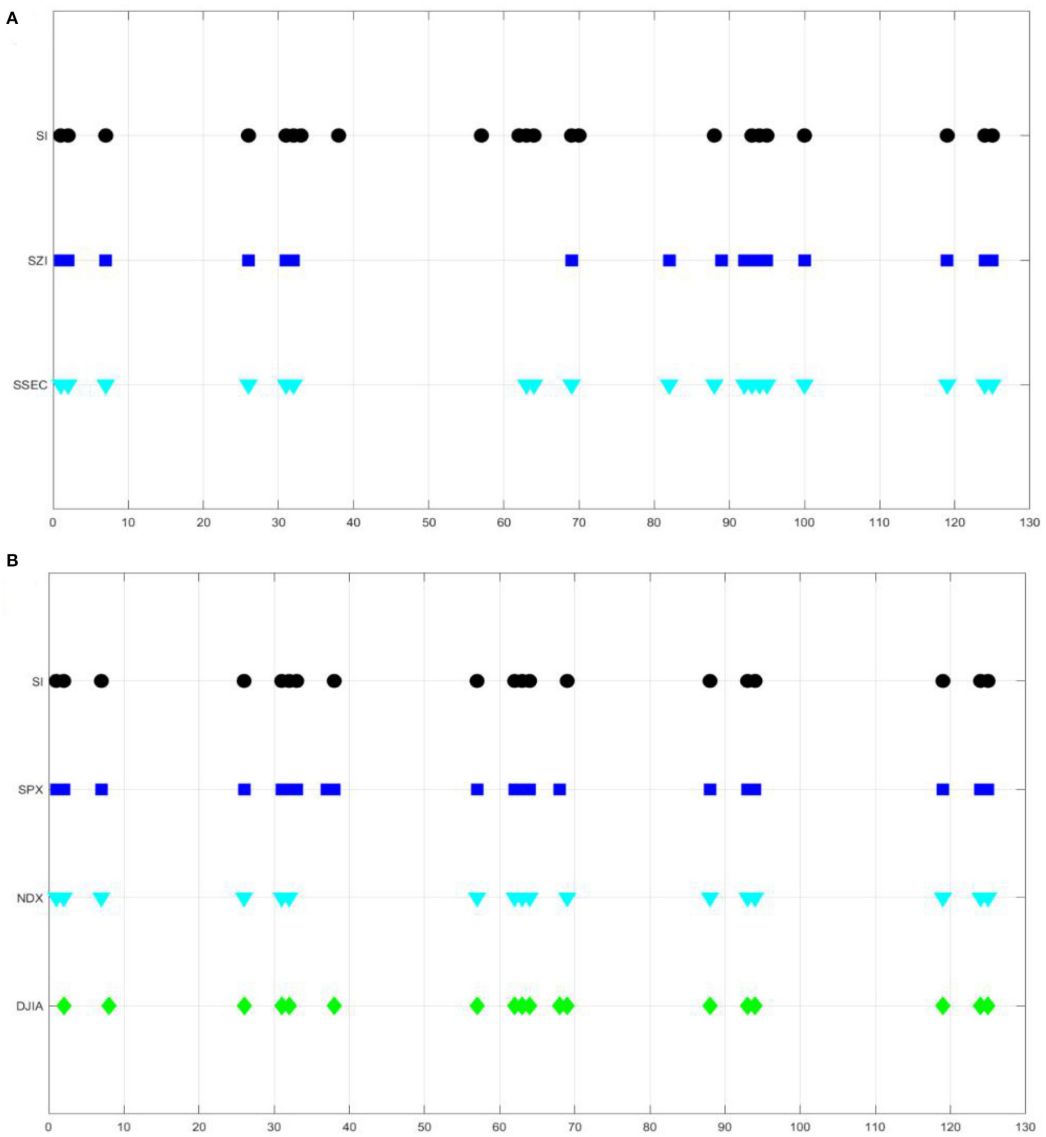
Quantization distribution of stock market indexes and investor sentiment indexes for both China and U.S. in terms of a vector graph with sub statuses. **(A)** China investor sentiment index, SZI and SSEC. **(B)** U.S. investor sentiment index, SPX, NDX, and DJIA.

In order to further potentially investigate the fundamental roles from the external elements of investors to the variability of the stock market, we have also made an attempt to systematically normalize and quantize all the stock market indexes and the investor sentiment indexes in both countries to integrate evidence into its common spatiotemporal responses and to help provide opportunities to detect the possible underlying patterns of stock market performances to be predicted under the circumstances of the COVID-19 pandemic. We first, respectively, normalized SSEC, SZI indexes, China Investor Sentiment Index, as well as NDX, SPX, DJIA indexes and U.S. Investor Sentiment Index, on a weekly average, to range from 0 to 1, then, for each given week, quantized all into five sub statuses and grouped every three consecutive elements along time domain as one vector in study. In this way, every weekly stock market index or investor sentiment index has been projected into a common three-dimensional vector space with a total of 125 sub statuses altogether. It is surprisingly demonstrated from our experiment results in Figure 5 that both the stock market indexes and investor sentiment indexes in each country followed nearly the homogenous spacial distribution in the vector graph after quantization. Although there is still much to be done in understanding topological attributes for such sophisticated spatial distributed models in stock markets, it did imply the strong consistency from the coarse-grained perspectives, when filtering those frequent fluctuations during the dynamic evolution of the COVID-19 pandemic. In our future studies, deep learning models, such as long-, short-term memory (LSTM), or complex networks, could be further established to develop the specific multivariate predictive model on the hypothesis, which might help insight into the status transition forces of stock market performance to be predicted and act as a substrate to topological properties, such as degree distributions, transitivity, self-similarity, global correlation, scale invariance, and community structure between stock market volatility and investor behaviors for such kind of public health events.

## Conclusion

As one of the black swan events in public health, COVID-19 brings unpredictable and unusual forces in the context of economics, and hereby results in a chain of adverse market reactions and disruptions. There has been a huge amount of sentiment swing among global investors, which, in turn, triggered a historic shakeout during the process. In this paper, we have quantitatively identified the time-frequency co-movement impact of COVID-19 on the U.S. and China stock markets since early 2020, NDX, DJIA, SPX, SSEC, and SZI in terms of the daily increase of COVID-19 confirmed cases across the daily closing prices across in favor of spatiotemporal interactions over investor sentiment index, and then proposed to explore the potential divisibility and the predictability to the volatility of the stock market during the evolution of COVID-19 over time. We have systemically retrieved the stock market variation and the growing number of COVID-19 cases in each country, and accordingly accessed and attained the natural logarithm—meanwhile, computed the weekly averaging to align with the investor sentiment indexes. To evaluate the time-frequency co-movement over COVID-19 impact, we have calculated the wavelet coherence map and the phase difference to capture the dominantly correlative phases and identify the leading roles COVID-19 performed along the time domain. We suggest the responses of stock market indexes in both countries to the COVID-19 epidemic in a long-term band, synchronized with the investor sentiment indexes, which could be roughly divided into three distinguished phases, namely, 30–75 (February 19, 2020-April 23, 2020), 110–150 (June 16, 2020-August 13, 2020), 220–280 (November 27, 2020-February 21, 2021) business days for China, and 80–125 (April 30, 2020-July 9, 2020) and 160–175 (August 27, 2020-September 17, 2020) after 290 (March 11, 2021) business days for U.S. At the first phase, the reason for the extreme volatility of the stock market mainly attributed to the sudden emergence of COVID-19 due to the pessimistic expectations from investors; the China and U.S. stock markets shared strongly negative correlation with COVID-19, resulting in the remarkably downward trend. The significant COVID-19 impact on Chinese economy has been recognized roughly 50 business days ahead, compared with the U.S. market, and the volatility of both stock markets was more correlated with its negative returns, and the epidemic was more likely to further exacerbate market panic, consistent with the leverage effect, exhibiting the strong asymmetries. At the second phase, the revitalization of the stock market shared strong simultaneous moves but interestingly exhibited quite opposite responses to the COVID-19 impact on the China and U.S. stock markets; the former retained a significant negative correlation between stock market indexes and the increase of COVID-19 cases, while the latter turned to positively correlated throughout the period. At the third phase, the progress in COVID-19 vaccine development and the economic stimulus began to impose forces to the stock market; the vulnerability to COVID-19 diminished to some extent as the investor sentiment indexes rebounded. Finally, we established a coarse-grained representation to the time series of both stock market and investor sentiment indexes, which demonstrated the homogenous spacial distribution in the vectorgraph after normalization and quantization, implying the strong consistency when filtering the frequent small fluctuations during the evolution of the COVID-19 pandemic. The proposed strategies could symmetrically provide the quantitative references in reasonably deducing the economic influences over public health issues. Although the developed scheme still tends to be an initial attempt to understand the volatility of the stock market, we believe it is quite reasonable to approximate the status transition, in accordance with the investor sentiment index, which might further help to have insights into the potential prediction in stock market performance.

## Data Availability Statement

Data of COVID-19 in China for this study are from is available the website of the National Health and Sanitation Commission of the People's Republic of China (http://www.nhc.gov.cn/). Data of COVID-19 in the United States is available from the Johns Hopkins Center for Systems Science and Engineering (JHU CSSE) (https://systems.jhu.edu/). The Shanghai Composite Index is available from the official website of Shanghai Stock Exchange (http://www.sse.com.cn/) and the Shenzhen Composite Index is available from the official website of Shenzhen Stock Exchange (http://www.szse.cn/). The Dow Jones Industrial Average, the S&P 500 Index, and the NASDAQ Index (National Association of Securities Dealers Automated Quotations Index) in the U.S. stock market and are available from Yahoo! Finance (https://finance.yahoo.com/). The SENSEX-30 Index in India Stock Market is from Investing Database (https://in.investing.com/). The China Investor Confidence Index is obtained from a survey conducted by the China Securities Investor Protection Fund, Ltd. and can be retrieved from the agency's official website (http://www.sipf.com.cn). The Sentix U.S. Investor Confidence Index can be retrieved from Sentix official website (https://www.sentix.de/).

## Author Contributions

RN has brought up the conception and the design of the study, guided the experiment work, together with the analysis and interpretation of data, drafted the manuscript, and made final consent about the manuscript to be published. YX has performed the experimental work and the acquisition of data, together with the analysis and interpretation of data, and drafted the manuscript. QY has assisted in the experimental work and the acquisition of data. CF provided help with the experiment and data analysis. AL has discussed the design of the study and helped with the experiment. All authors contributed to the article and approved the submitted version.

## Funding

This work was supported by the National Social Science Fund of China (20BJY021).

## Conflict of Interest

QY was employed by company Suning Software Technology Co., Ltd., Nanjing, China. The remaining authors declare that the research was conducted in the absence of any commercial or financial relationships that could be construed as a potential conflict of interest.

## Publisher's Note

All claims expressed in this article are solely those of the authors and do not necessarily represent those of their affiliated organizations, or those of the publisher, the editors and the reviewers. Any product that may be evaluated in this article, or claim that may be made by its manufacturer, is not guaranteed or endorsed by the publisher.
